# Nationwide analysis of inpatient laparoscopic ventral hernia repair in Italy from 2015 to 2020

**DOI:** 10.1007/s13304-023-01460-4

**Published:** 2023-03-14

**Authors:** Emanuele Botteri, Monica Ortenzi, Sophie Williams, Andrea Balla, Mauro Podda, Mario Guerrieri, Alberto Sartori

**Affiliations:** 1 General Surgery, ASST Spedali Civili di Brescia PO Montichiari, Via Boccalera, 325018 Montichiari, Brescia Italy; 2 Department of General and Emergency Surgery, Università Politecnica Delle Marche, Piazza Roma 22, 60121 Ancona, Italy; 3 Colorectal Department, King’s College Hospital, London, UK; 4 UOC of General and Minimally Invasive Surgery, Hospital “San Paolo”, Largo Donatori del Sangue 1, 00053 Civitavecchia, RM Italy; 5 Department of Surgical Science, University of Cagliari, Cagliari, Italy; 6 Department of General Surgery, Ospedale di Montebelluna, Via Palmiro Togliatti, 16, 31044 Montebelluna, TV Italy

**Keywords:** Ventral hernia, Laparoscopic, Nationwide analysis

## Abstract

Since 2010, several guidelines and consensus papers have been proposed to support surgeons in the decision-making process (Cuccurullo et al. in Hernia 17(5):557–566, 2013; Silecchia et al. in Surg Endosc 29:2463–2484, 2015; Bittner et al. in Surg Endosc 33(11):3511–3549, 2015) with the conclusion that laparoscopic repair (LR) has gained popularity in the treatment of IH.

To date, however, it is not yet clear as to the uptake of LR for IH on national basis. Only dated studies encompassing of all types of incisional hernia repairs are available in literature (Bisgaard et al. in Br J Surg 96:1452–1457, 2009). The aim of our study is to present a snapshot of Italian data for LR of ventral hernias, over a 6 years period, including volume of LR, procedural features and major postoperative outcomes. Data were extracted from the Italian Hospital Information System (HIS) that collects clinical and administrative information regarding each hospital admission of every patient discharged from any hospital in Italy. Using Hospital Discharge records regional Databases (HDD), all laparoscopic ventral hernia procedures carried out in public and private hospitals between 2015 and 2020, in patients over 18 years and resident in Italy, were collected based on diagnosis and procedure codes. The National Agency for Regional Health Services (AgeNaS) oversees the management and analysis of data. All hospital admissions that occurred between 2015 and 2020 were analyzed.

A total of 154,546 incisional hernia repairs were performed in Italy from 2015 to 2020. Of these, 20,789 (13.45%) were minimally invasive repairs. The number of procedures performed increased significantly over time, constituting 11.96 and 15.24% of all procedures performed in 2015 and 2020 respectively. However, considering the whole period, the mean annual change was—5.58% (CI − 28.6% to 17.44%; *p* < 0.0001).

Urgent minimally invasive repairs were performed in 1968 cases (1.27%). The absolute rate of laparoscopically treated patients needing an urgent surgical procedure increased overtime (from 7.36% in 2015 to 13.418% in 2020). The mean annual change registered over the whole period was 7.42%. 92% (CI − 0.03 to 14.09%; *p* < 0.0001). However, when considering the period from 2015 to 2019, the mean annual change was 10.42% (CI 6.35 to 14.49%; *p* < 0.0001). To our knowledge this is the first nationwide Italian report presenting the national workload of surgical units and the main perioperative features of minimally invasive surgery for ventral hernia repairs.

**Supplementary Information:**

The online version contains supplementary material available at 10.1007/s13304-023-01460-4.

## Introduction

Following abdominal surgery, incisional hernias (IH) are associated with 20% of procedures, with a global lifetime risk of 5% [1]. The healthcare system costs for the treatment of ventral hernias are high and these costs are expected to continue to rise [2]. There are a number of approaches to incisional hernia repair, in addition to traditional open methods, including, several endoscopic, laparoscopic and hybrid procedures.

Since 2010, several guidelines and consensus papers have been proposed to support surgeons in the decision-making process [3–5] with the conclusion that laparoscopic repair (LR) has gained popularity in the treatment of IH.

To date, however, it is not yet clear as to the uptake of LR for IH on national basis. Only dated studies encompassing of all types of incisional hernia repairs are available in literature [6].

The aim of our study is to present a snapshot of Italian data for LR of ventral hernias, over a six years period, including volume of LR, procedural features and major postoperative outcomes.

## Materials and methods

### Data extraction

Data were extracted from the Italian Hospital Information System (HIS) that collects clinical and administrative information regarding each hospital admission of every patient discharged from any hospital in Italy. Using Hospital Discharge records regional Databases (HDD), all laparoscopic ventral hernia procedures carried out in public and private hospitals between 2015 and 2020, in patients over 18 years and resident in Italy, were collected based on diagnosis and procedure codes.

The National Agency for Regional Health Services (AgeNaS) oversees the management and analysis of data. All hospital admissions that occurred between 2015 and 2020 were analyzed. Subsequently, information on every admission with a diagnosis of incisional hernia as either primary or secondary [ICD-9-CM code 5512x, 5522x, 5532x,] were extracted. The presence of intestinal obstruction at the time of the operation was also registered [ICD-9-CM code 5518, 5528, 5529].

### General data

Gender, age and preexisting comorbidities were considered. As for the latter they were divided into general comorbidities [ICD-9-CM code atrial fibrillation (42,731), diabetes (2500×); anticoagulant therapy (2865 × and V5861) respiratory failure (49,120), renal failure (5859×) and obesity (278.0×).

Complication rate was also collected, either as primary diagnosis or among the first five secondary diagnosis [ICD-9-CM code bleeding (99,811), haematoma (99,812), serohematoma (99,813); infection (9960×–9965×), wound (998.59 e 9989×), bowel (99,687)] additionally, the presence of colostomy or ileostomy [ICD-9-CM V44.3 V44.2] was registered.

### Procedural data

In this study, the discrimination between laparoscopic or open incisional hernia repair was defined as the presence or the absence of the ICD-9-CM code 54.21 as secondary diagnosis while robotic-assisted procedure was classified with the ICD-9-CM code 0039. The conversion to open surgery from minimally invasive surgery has a separate ICD-9-CM codification (6411). The association with other surgical procedures was also considered.

Based on HIS data and patient discharge records, the analysis included total hospital stay (primary plus readmission within 30 days), readmission rate, early mortality (within the first admission and < 30 days from the operation), late mortality (> 30-day mortality) and 30-day morbidity.

### Statistical analysis

Data were processed using the MedCalc statistical package (version 12.5). Qualitative variables were summarized by frequency and percentage, while non-normally distributed quantitative variables were described by the median and standard deviation (SD).

Statistical analysis was performed using Student’s t test and the Cochran Armitage test for trend as appropriate. A two-tailed *p* value < 0.05 was considered statistically significant. The sample size was the Italian population, reported by region, according to the average yearly population for the period 2015–2020, reported by the Italian National Institute of Statistics (ISTAT) (Supplemental Table S1).

## Results

A total of 154,546 incisional hernia repairs were performed in Italy from 2015 to 2020. Of these, 20,789 (13.45%) were minimally invasive repairs.

The number of procedures performed increased significantly over time, constituting 11.96 and 15.24% of all procedures performed in 2015 and 2020 respectively. However, considering the whole period, the mean annual change was − 5.58% (CI − 28.6 to 17.44%; *p* < 0.0001).

**Fig. 1:**
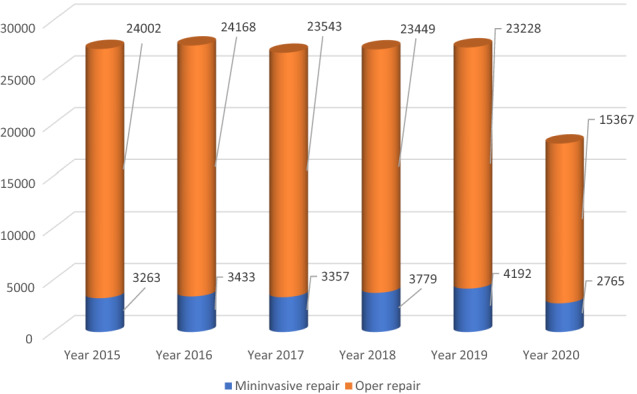
Open and minimally invasive ventral hernia repairs in absolute and relative frequencies performed in the index period (source AGENAS)

Specifically, a negative trend was observed between 2016 and 2017 (– 2.65%), and between 2019 and 2020 was − 51, 60%. However, if considering the period between 2015 and 2019 the mean annual change was 5.92% (CI − 0.03 to 11.87%; *p* < 0.0001) Fig. 1.

A significant increase was also registered in the number of ventral hernias performed robotically, with a mean annual change of 9.37% (CI − 10.22 to 28.96%; *p* < 0.0001) between 2015 and 2020 and of 36.71% (CI 28.15 to 45.27%; *p* < 0.0001) when considering only the first five years.

The gender ratio was about 44% for male patients for the entire period without significant fluctuation overtime and the mean age of treated patients ranged from 61 ± 13 in 2015 to 63 ± 13 in 2020.

### Procedural data

**Fig. 2:**
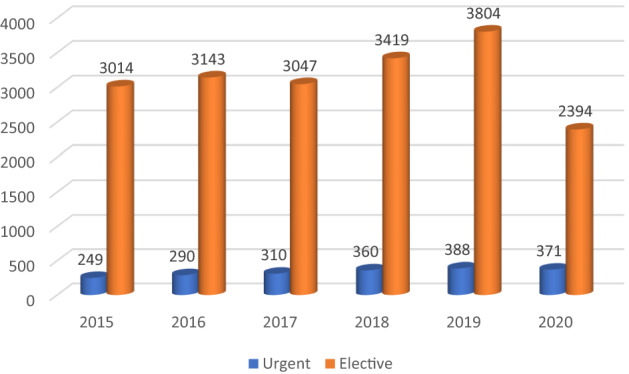
Elective and urgent procedures in absolute values (source AGENAS)

Urgent minimally invasive repairs were performed in 1968 cases (1.27%). The absolute rate of laparoscopically treated patients needing an urgent surgical procedure increased overtime (from 7.36% in 2015 to 13.418% in 2020). The mean annual change registered over the whole period was 7.42% 0.92% (CI: − 0.03 to 14.09%; *p* < 0.0001). However, when considering the period from 2015 to 2019 the mean annual change was 10.42% (CI 6.35 to 14.49%; *p* < 0.0001) Fig. 2.

**Fig. 3:**
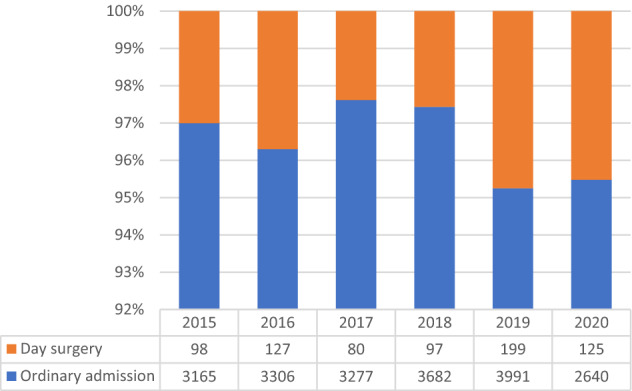
Type of hospital admission in absolute values in the index period (source AGENAS)

A total of 7714 (37.11%) incisional hernia repairs were associated with other surgical procedures during this period. The most performed concurrent procedure was minimally invasive adhesiolysis, here, a slight increase was observed from 2015 to 2020 (mean annual change = 6.75%; CI 5.1 to 8.34%; *p* = 0.445), while the mean annual change for the whole period was − 4.64% (CI − 27.55 to 18.27%; *p* = 0.542) (Fig. 3).

### Type of admission and hospital stay

The number of patients treated as elective admissions ranged from 2640 in 2020 to 3991 in 2019 as absolute numbers and from 95.21% to 97.43% as relative values to overall procedures in 2019 and 2017 respectively. The mean annual change was -5.81 (CI − 28.37 to 16.75%; *p* < 0.0001), however, a positive mean annual change was observed if considering only the first five years (mean annual change = 5.53%; CI 10.51 to 0.55%; *p* = 0.003).

The number of procedures managed as day case procedures ranged from 80 in 2020 to 199 in 2019 as absolute numbers and from 2.38 to 4.75% as rates of overall procedures in 2017 and 2019 respectively. Overall, the mean annual change was − 5.27 (CI − 46.69 to 39.15%; *p* < 0.0001), however, a positive mean annual change was observed if considering only the first five years (8.22%; CI − 37.87 to 54.31%; *p* = 0.003).

Mean hospital stay was 5 days in 2015, 2018 and 2020. In 2016, 2017, 2019 it was 4 days*.*

### Conversion, complications and readmission

Overall, a total of 271 (1.3%) conversions, 193 (0.93%) complications and 58 (0.28%) readmissions within 30 days were registered. Conversion rate significantly increased over the considered period with a mean annual change of 10.10% (CI − 4.64 to 24.84%; *p* < 0.0001) from 2015 to 2020 and of 13.12% 5.27 (CI − 4.31 to 30.55%; *p* = 0.0322). Complications ranged from 26 in 2016 to 40 in 2017 as absolute numbers and from 0.87 to 1.19% as rate of overall procedures in 2017 and 2019 respectively (mean annual change = − 3.5%; CI − 25.92 to 18.92%; *p* = 0.911). Readmissions ranged from 6 in 2020 to 15 in 2019, (mean annual change = − 20.44%; CI −93.84 to 52.96%; *p* = 0.487 from 2015 to 2020; mean annual change = 11.95%; CI − 35.62 to 59.52%; *p* = 0.184 from 2015 to 2019) Fig. 4.

**Fig. 4:**
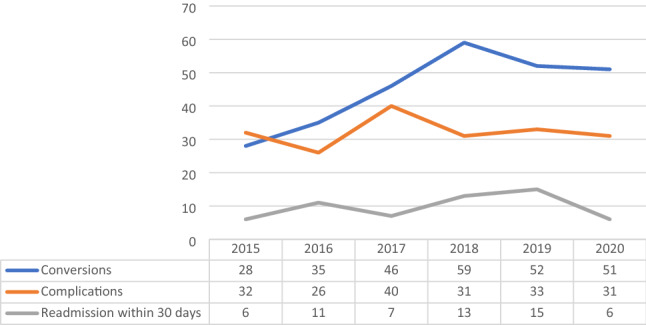
Complications, conversions and readmissions within 30 days rates (source AGENAS)

### Mortality rate

A total of 60 (0.28%) deaths were registered (mean annual change = 1.07%; CI − 37.64 to 39.78%; *p* = 0.142 from 2015 to 2020; mean annual change = − 7.58%; CI − 52.5 to 37.34; *p* = 0.887 from 2015 to 2019).

Thirty-two (53.33%) deaths occurred within 30 days from the operations and 28 after 30 days (46.67%). The mean annual change for early mortality was − 38.94% and − 40.34% with and without considering 2020 respectively (CI − 115.73 to 37.85%; *p* = 0.655 and CI − 139.42 to 58.78%; *p* = 0.943, respectively). The mean annual change for late mortality was -5.09% and -20% with and without considering 2020 respectively (CI − 87.13 to 76.95%; *p* = 0.008 and CI − 64.92 to 24.92%; *p* = 0.744, respectively).

## Regional data

The number of elective procedures performed laparoscopically steadily increased across Italy, however, in six regions, the difference was not significant considering both the whole period and the first five years without analyzing 2020 data (Valle d’Aosta, Trentino, Friuli Venezia Giulia, Toscana, Umbria Abruzzo Molise and Campania). The minimum annual intervention rate was observed in Calabria (AIR = 1) while the maximum was registered in Valle d’Aosta (19 in 2018). Table 1

**Table 1:** Regional data about elective laparoscopic ventral hernia repairs in the index period

Region	Year					
	2015	2016	2017	2018	2019	2020
A. Annual Interventions Rate (AIR) for elective laparoscopic ventral hernia repairs (100,000 inhabitants) in Italy from 2015 to 2020 (sources Agenas and Italian National Institute of Statistics (2022) Resident population on 31st December. ISTAT. http://dati.istat.it/?lang=en#)						
Piemonte	4	5	4	5	7	4
Valle d’aosta	9	11	13	19	11	7
Lombardia	7	7	7	8	9	5
Trentino alto adige	8	10	6	7	7	5
Veneto	8	8	9	10	9	7
Friuli venezia giulia	7	9	5	7	8	5
Liguria	7	6	4	4	5	3
Emilia-romagna	11	10	10	12	12	9
Toscana	7	8	7	8	9	6
Umbria	15	14	10	14	17	8
Marche	5	7	10	16	14	8
Lazio	4	4	4	4	5	5
Abruzzo	3	6	8	4	6	6
Molise	5	5	7	2	3	3
Campania	2	2	2	2	3	2
Puglia	3	3	3	4	4	3
Basilicata	2	3	4	3	4	3
Calabria	3	2	1	1	2	2
Sicilia	2	3	3	3	4	3
Sardegna	4	4	6	5	6	4

Region	Year						
	2015	2016	2017	2018	2019	2020	Total
B. Annual absolute numbers for elective laparoscopic ventral hernia repairs in Italy from 2015 to 2020							
Piemonte	194	211	193	222	287	173	1280
Valle d’Aosta	11	14	17	24	14	9	89
Lombardia	680	735	662	780	926	467	4250
PA di Bolzano	33	25	23	17	19	12	129
PA di Trento	53	82	59	62	58	37	351
Veneto	379	381	447	468	457	324	2456
Friuli Venezia Giulia	88	114	64	89	102	55	512
Liguria	115	95	65	64	74	41	454
Emilia-Romagna	471	450	463	543	541	405	2873
Toscana	270	282	266	280	329	216	1643
Umbria	134	127	90	122	147	65	685
Marche	84	100	160	248	218	118	928
Lazio	226	207	211	249	268	259	1420
Abruzzo	43	75	101	52	75	73	419
Molise	16	15	21	5	8	10	75
Campania	133	141	113	113	172	121	793
Puglia	102	123	115	156	148	113	757
Basilicata	9	17	22	19	24	17	108
Calabria	49	40	19	27	34	33	202
Sicilia	107	137	151	152	198	154	899
Sardegna	66	62	95	87	93	63	466

Region	YEAR					*p*	*p*^*1*^
	2016	2017	2018	2019	2020		
C. Annual trend in the index period							
Piemonte	8.06	− 9.33	13.06	22.65	− 65.90	< 0.0001	< 0.0001
Valle d'aosta	21.43	17.65	29.17	− 71.43	− 55.56	0.768	0.386
Lombardia	7.48	− 11.03	15.13	15.77	− 98.29	< 0.0001	< 0.0001
Pa di bolzano	− 32.00	− 8.70	− 35.29	10.53	− 58.33	0.0004	0.0009
Pa di trento	35.37	− 38.98	4.84	− 6.90	− 56.76	0.102	0.054
Veneto	0.52	14.77	4.49	− 2.41	− 41.05	0.0006	0.0001
Friuli venezia giulia	22.81	− 78.13	28.09	12.75	− 85.45	0.961	0.683
Liguria	− 21.05	− 46.15	− 1.56	13.51	− 80.49	0.0025	0.0011
Emilia-romagna	− 4.67	2.81	14.73	− 0.37	− 33.58	< 0.0001	< 0.0001
Toscana	4.26	− 6.02	5.00	14.89	− 52.31	0.007	0.0612
Umbria	− 5.51	− 41.11	26.23	17.01	− 126.15	0.681	0.130
Marche	16.00	37.50	35.48	− 13.76	− 84.75	< 0.0001	< 0.0001
Lazio	− 9.18	1.90	15.26	7.09	− 3.47	< 0.0001	0.0422
Abruzzo	42.67	25.74	− 94.23	30.67	− 2.74	0.0018	0.083
Molise	− 6.67	28.57	− 320.00	37.50	20.00	0.586	0.064
Campania	5.67	− 24.78	0.00	34.30	− 42.15	0.0155	0.383
Puglia	17.07	− 6.96	26.28	− 5.41	− 30.97	< 0.0001	0.0006
Basilicata	47.06	22.73	− 15.79	20.83	− 41.18	0.0008	0.0109
Calabria	− 22.50	− 110.53	29.63	20.59	− 3.03	0.268	0.0143
Sicilia	21.90	9.27	0.66	23.23	− 28.57	< 0.0001	< 0.0001
Sardegna	− 6.45	34.74	− 9.20	6.45	− 47.62	0.0005	0.0002

*p*^*1*^ Cochrane Hermitage results without considering 2020

Considering urgent procedures, an increase in the adoption of laparoscopy was observed, however, in 9 regions, this increase was not significant considering both the whole period and the first five years excluding the 2020 data (Valle d’Aosta, Trentino, Veneto, Liguria, Umbria, Abruzzo, Molise, Basilicata, Calabria). Many regions showed the same annual intervention rate (AIR = 0) while the maximum was registered in Trentino [3]. Table 2 summarizes the distribution of urgent procedures in the index period.

**Table 2:** Regional data about urgent laparoscopic ventral hernia repairs in the index period

Region	Year					
	2015	2016	2017	2018	2019	2020
A. Annual Interventions Rate (AIR) for urgentlaparoscopic ventral hernia repairs (1,000,000 inhabitants) in Italy from 2015 to 2020 (sources Agenas and Italian National Institute of Statistics (2022) Resident population on 31st December. ISTAT. http://dati.istat.it/?lang=en#.)						
Piemonte	4	2	5	3	6	4
Valle d’aosta	16	8	8	40	48	8
Lombardia	4	5	6	6	6	5
Trentino alto adige	4	8	9	7	6	6
Veneto	2	7	5	7	7	8
Friuli venezia giulia	2	2	1	7	4	7
Liguria	10	8	4	5	6	6
Emilia-romagna	8	9	10	11	9	12
Toscana	5	8	7	11	8	8
Umbria	11	18	16	16	24	8
Marche	4	4	10	18	15	13
Lazio	5	4	3	4	4	5
Abruzzo	3	3	2	6	7	5
Molise	0	3	3	0	0	10
Campania	2	2	2	2	4	3
Puglia	2	2	4	5	8	8
Basilicata	4	7	12	5	11	11
Calabria	3	2	2	4	1	2
Sicilia	2	4	4	4	6	6
Sardegna	2	3	5	3	4	6

Region	YEAR						
	2015	2016	2017	2018	2019	2020	Total
B. Annual absolute numbers for urgent laparoscopic ventral hernia repairs in Italy from 2015 to 2020							
Piemonte	17	9	21	15	27	16	105
Valle d’aosta	2	1	1	5	6	1	16
Lombardia	44	51	56	58	61	48	318
Pa di bolzano	2	1	4	3	2	3	15
Pa di trento	2	8	6	4	4	3	27
Veneto	12	32	26	32	32	41	175
Friuli venezia giulia	3	3	1	8	5	9	29
Liguria	15	12	6	8	9	9	59
Emilia-romagna	37	41	45	48	40	52	263
Toscana	18	31	27	42	28	28	174
Umbria	10	16	14	14	21	7	82
Marche	6	6	16	27	23	19	97
Lazio	31	22	17	21	23	30	144
Abruzzo	4	4	3	8	9	6	34
Molise	0	1	1	0	0	3	5
Campania	14	13	14	14	24	19	98
Puglia	8	7	15	20	32	30	112
Basilicata	2	4	7	3	6	6	28
Calabria	6	4	4	7	2	4	27
Sicilia	12	19	18	18	27	28	122
Sardegna	4	5	8	5	7	9	38
Total	249	290	310	360	388	371	1968

Region	Annual trend					*p*	*p*^*1*^
	2016	2017	2018	2019	2020		
C. Annual trend in the index period							
Piemonte	− 88,89	57,14	− 40,00	44,44	− 68,75	< 0.0001	< 0.0001
Valle d’aosta	− 100,00	0,00	80,00	16,67	− 500,00	0.768	0.386
Lombardia	13,73	8,93	3,45	4,92	− 27,08	< 0.0001	< 0.0001
Pa di bolzano	− 100,00	75,00	− 33,33	− 50,00	33,33	0.0004	0.0009
Pa di trento	75,00	− 33,33	− 50,00	0,00	− 33,33	0.1026	0.0546
Veneto	62,50	− 23,08	18,75	0,00	21,95	0.0006	0.0001
Friuli venezia giulia	0,00	− 200,00	87,50	− 60,00	44,44	< 0.0001	0.6838
Liguria	− 25,00	− 100,00	25,00	11,11	0,00	0.0025	0.0011
Emilia-romagna	9,76	8,89	6,25	− 20,00	23,08	< 0.0001	< 0.0001
Toscana	41,94	− 14,81	35,71	− 50,00	0,00	0.0070	0.0612
Umbria	37,50	− 14,29	0,00	33,33	− 200,00	0.6814	0.1302
Marche	0,00	62,50	40,74	− 17,39	− 21,05	< 0.0001	< 0.0001
Lazio	− 40,91	− 29,41	19,05	8,70	23,33	< 0.0001	0.0422
Abruzzo	0,00	− 33,33	62,50	11,11	− 50,00	0.0018	0.0838
Molise	100,00	0,00	0,00	0,00	100,00	0.2813	0.0641
Campania	− 7,69	7,14	0,00	41,67	− 26,32	0.0070	0.2451
Puglia	− 14,29	53,33	25,00	37,50	− 6,67	< 0.0001	0.0006
Basilicata	50,00	42,86	− 133,33	50,00	0,00	0.0008	0.0109
Calabria	− 50,00	0,00	42,86	− 250,00	50,00	0.2683	0.0143
Sicilia	36,84	− 5,56	0,00	33,33	3,57	< 0.0001	< 0.0001
Sardegna	20,00	37,50	− 60,00	28,57	22,22	0.0005	0.0002

*p*^*1*^ Cochrane Hermitage results without considering 2020

## Discussion

The indications and limitations for laparoscopic ventral hernia repair are well established by different guidelines [3–5], some of which were published before 2015.

In Italy, between 2015 and 2019 more than 26,000 patients underwent surgery for ventral hernia repair. The number of laparoscopic ventral hernia procedures in this period reached over 3000 patients annually, with a significant increase in its adoption from 2015 to 2019 (Table 3).

**Table 3:** Annual trend of open and laparoscopic hernia repairs (source AGENAS)

% Open vs laparoscopic incisional hernia repair						
	2015	2016	2017	2018	2019	2020
Laparoscopy (%)	11.97	12.44	12.48	13.88	15.29	15.25
Open (%)	88.00	87.56	87.52	86.12	84.71	84.75

Annual trend						
	2015	2016	2017	2018	2019	2020
Laparoscopy (%)	x	4.95	− 2.26	11.70	9.85	− 51.61
Open (%)	x	0.69	− 2.65	− 0.40	− 0.95	− 51.16

2015–2020			
	Mean	SD	± CI 95%
Laparoscopy (%)	− 5.58	26.26	23.01
Open (%)	− 10.89	22.53	19.76

2015–2019			
	Mean	SD	± CI 95%
Laparoscopy (%)	5.93	6.08	5.95
Open (%)	− 0.83	1.39	1.36

*SD* standard deviation, *CI* confidence interval

However, even if this positive trend is confirmed in the future, thanks to the dissemination of several interesting new techniques [5] the indications for minimally invasive ventral hernia repair are likely to expand; indeed, in 2019 and 2020 the laparoscopic approach stood at 15% of the total number of ventral repairs (Table 4).

**Table 4:** Annual trend of urgent incisional hernia repairs (source AGENAS)

Urgent laparoscopic incisional hernia repair						
	2015	2016	2017	2018	2019	2020
Urgent (%)	7.63	8.45	9.23	9.52	9.26	13.14
Annual trend (%)	x	14.14	6.45	13.89	7.26	-4.50

Trend period 2015–2020			
	Mean	SD	± CI 95%
Urgent (%)	7.42	7.62	6.68

Trend period 2016–2019			
	Mean	SD	± CI 95%
Urgent (%)	10.42	4.16	4.07

*SD* standard deviation, *CI* confidence interval

As COVID-19 spread in 2020, Italy, along with the rest of the world, faced an unexpected new healthcare emergency which significantly affected surgical activity. [7].

Elective surgery was particularly affected by the pandemic, with numbers of elective procedures for benign conditions, dramatically reduced [8].

In 2020, the number of ventral repairs, both open and minimally invasive, almost halved if compared to 2019. Naturally, this trend affected observations that could be made regarding the adoption of minimally invasive repair in Italy over the index period, therefore, we opted to split the statistical analysis, by considering the whole period as well as omitting the 2020 data.

Nevertheless, an interesting observation is that, despite the dramatic drop in the number of performed procedures in 2020, [9, 10] the rate of minimally invasive procedures across the total number of procedures performed remained unchanged from 2019 to 2020, with minimally invasive procedures accounted for almost 15% of all ventral hernia repairs performed in these two years.

The impact of Sars-Cov-2 on elective surgical activity is more evident when analyzing data related to urgent procedures: the decrease in absolute numbers observed in 2020 was also noted in the number of urgent ventral hernia repairs, however, urgent procedures in 2020 represented the 13.14% of the whole number of ventral hernia repairs performed in 2020, in contrast to 2019 when urgent procedures represented the 9.26% of the total procedures.

In the literature, urgent repair is not a contraindication to laparoscopy, however, laparoscopy is generally contraindicated in the presence of diffuse peritonitis or marked bowel distension [11–15] in addition to the general advise against the use of pneumoperitoneum in cases of hemodynamic instability or severe lung and heart disease [15, 16]. Our study confirms that laparoscopic ventral hernia repair is safe, with a complication rate of 0.93% and a percentage of readmission within 30 days of 0.4%. There is sufficient data presented in literature based on several meta-analyses and randomized controlled trials to support laparoscopic mesh repair for IH as a low risk procedure [17–20]. Most laparoscopic IH repairs require an elective admission with a mean hospital stay of 4–5 days. Type of hospital admission and length of stay are strongly influenced by mesh size, patient age and comorbidities. Complex defects and incarcerated hernias are also predictive factors associated with longer hospitalization [21].

Regional data showed a high variability among the Italian regions in the adoption of the laparoscopic approach when dealing with incisional hernias. This might be explained by the fact that the adoption of the laparoscopic approach is mostly based on the performing surgeons’ preferences and training and on the actual resources of the hospitals. In our opinion, this observation may highlight the urgency for standardization for this procedure and its indications as well as for the publication of proper guidelines.

## Conclusions

To our knowledge, this is the first nationwide Italian report presenting the national workload of surgical units and the main perioperative features of minimally invasive surgery for ventral hernia repairs.

The definition of ventral hernia encompasses several hernia types, differing in size of the fascial defect and number and complexity of the previous surgical procedures. The coding system is not able to discriminate accurately the nature of all ventral hernias, therefore, the results of the present work, particularly where open repair is compared, could be influenced by bias. Still, there was a steady increase in the adoption of the laparoscopic approach in both emergent and elective settings in the index period. The high variability observed among the Italian regions.

## Supplementary Information

Below is the link to the electronic supplementary material.

Supplementary file1 (DOCX 19 KB)

Supplementary file2 (DOCX 917 KB)

Supplementary file3 (DOCX 928 KB)

Supplementary file4 (DOCX 619 KB)

## Data Availability

Data will be available if requested.
