# Supplementary material for: Nationwide analysis of inpatient laparoscopic ventral hernia repair in Italy from 2015 to 2020
Source: Updates Surg. 2023 Mar 14;75(6):1661–70. doi: 10.1007/s13304-023-01460-4 (PMC10013272; doi:10.1007/s13304-023-01460-4)
Supplement: Supplementary file 2 — Supplementary file2 (DOCX 917 KB) [file 13304_2023_1460_MOESM2_ESM.docx]

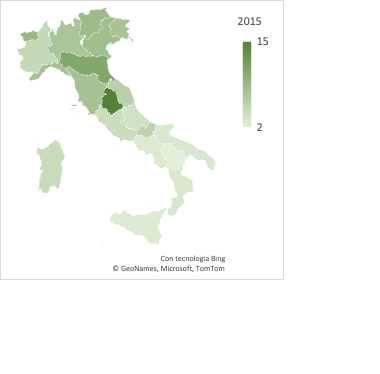

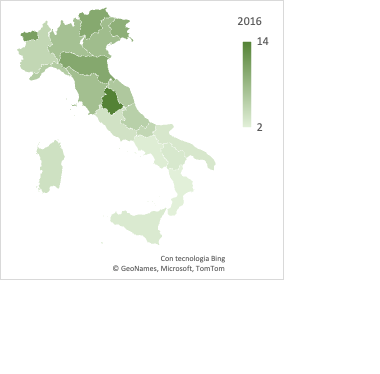


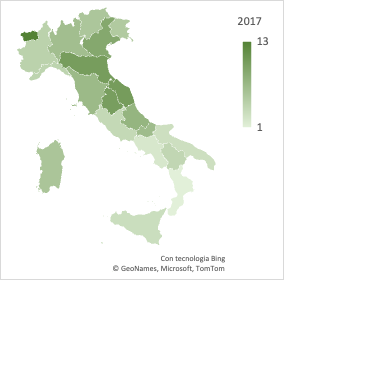

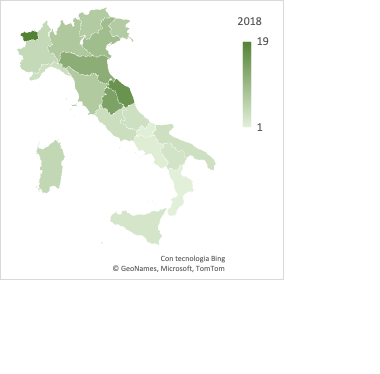

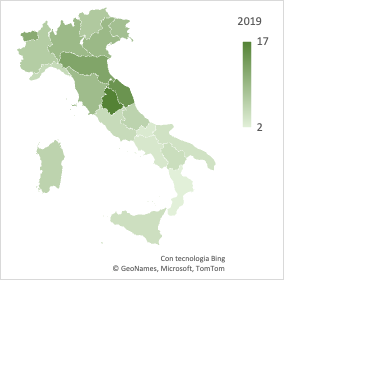

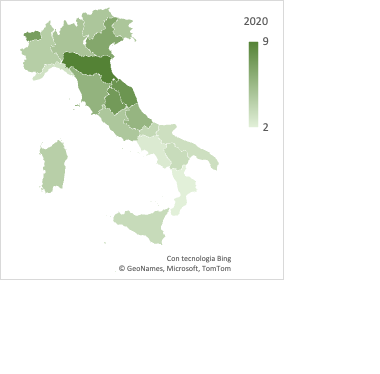


***Supplemental Figure 1***Annual Interventions Rate (AIR) for elective laparoscopic ventral hernia procedures (100,000 inhabitants) in Italy from 2015 to 2020 (sources Agenas and Italian National Institute of Statistics (2022) Resident population on 31st December. ISTAT. <http://dati.istat> .it/?lang=en#.)
