# Supplementary material for: Nationwide analysis of inpatient laparoscopic ventral hernia repair in Italy from 2015 to 2020
Source: Updates Surg. 2023 Mar 14;75(6):1661–70. doi: 10.1007/s13304-023-01460-4 (PMC10013272; doi:10.1007/s13304-023-01460-4)
Supplement: Supplementary file 3 — Supplementary file3 (DOCX 928 KB) [file 13304_2023_1460_MOESM3_ESM.docx]

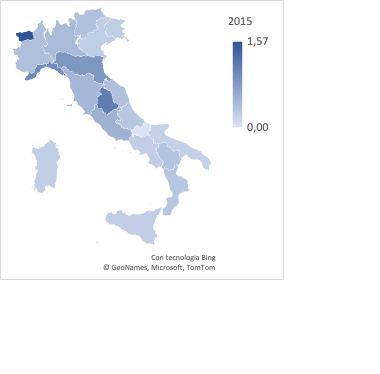

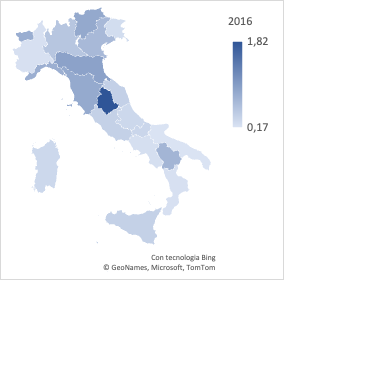


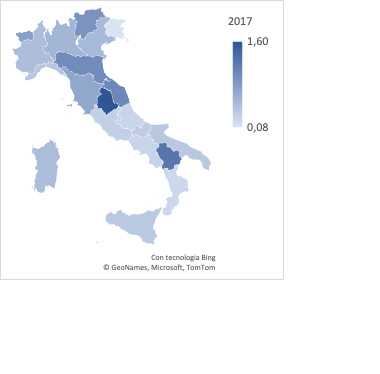

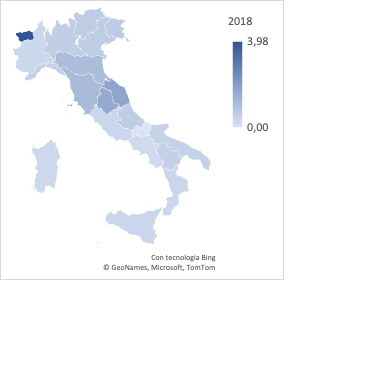


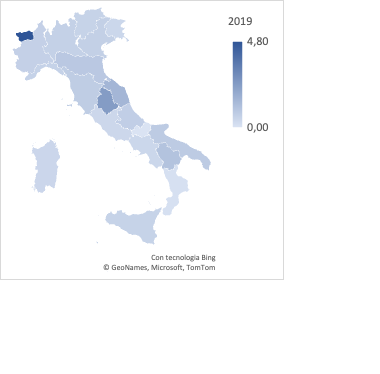

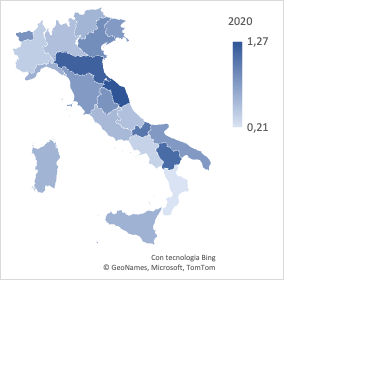


***Supplemental Figure 2*** Annual Interventions Rate (AIR) for urgent laparoscopic ventral hernia procedures (100,000 inhabitants) in Italy from 2015 to 2020 (sources Agenas and Italian National Institute of Statistics (2022) Resident population on 31st December. ISTAT. <http://dati.istat> .it/?lang=en#.)
