# Supplementary material for: Nationwide analysis of inpatient laparoscopic ventral hernia repair in Italy from 2015 to 2020
Source: Updates Surg. 2023 Mar 14;75(6):1661–70. doi: 10.1007/s13304-023-01460-4 (PMC10013272; doi:10.1007/s13304-023-01460-4)
Supplement: Supplementary file 4 — Supplementary file4 (DOCX 619 KB) [file 13304_2023_1460_MOESM4_ESM.docx]

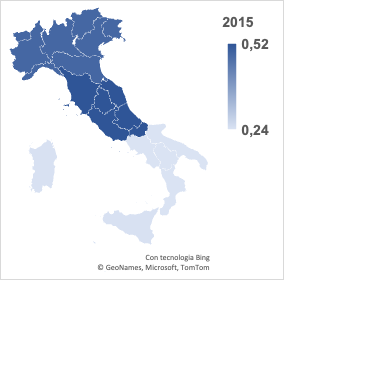

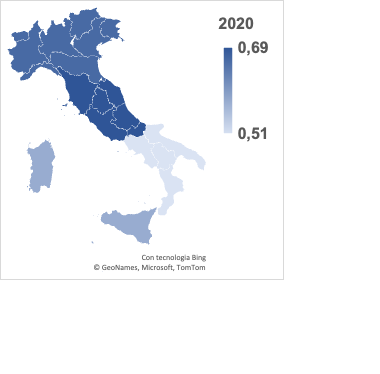
 A


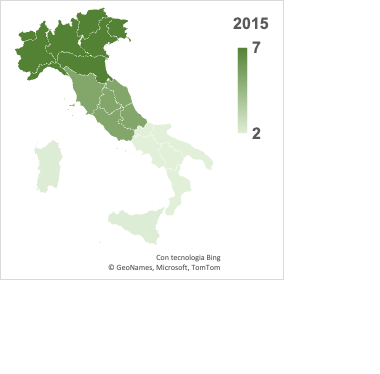

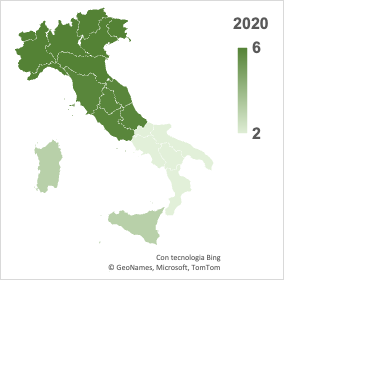
 B

***Supplemental Figure 3*** AIR of elective (A) and urgent (B) laparoscopic ventral hernia procedures (100,000 inhabitants) in Italian population divided in microregion in 2015 and 2020 (sources Agenas and Italian National Institute of Statistics (2019) Resident population on 31st December. ISTAT. <http://dati.istat> .it/?lang=en#.)
